# Development and Implementation of a Defect Detection Model for Microstructures Using Image Processing Methods

**DOI:** 10.3390/ma18225207

**Published:** 2025-11-17

**Authors:** Sandra Gajoch, Dorota Wilk-Kołodziejczyk, Łukasz Marcjan, Roberto Corizzo, Adam Bitka, Marcin Małysza, Gerard Skomin

**Affiliations:** 1 Department of Applied Computer Science and Modelling, Faculty of Metals Engineering and Computer Science, AGH University of Krakow, al. Adama Mickiewicza 30, 30-059 Krakow, Poland; lmarcjan@agh.edu.pl (Ł.M.); gerardskomin@student.agh.edu.pl (G.S.); 2 Lukasiewicz Research Network-Krakow Institute of Technology, Zakopiańska 73, 30-418 Kraków, Poland; adam.bitka@kit.lukasiewicz.gov.pl (A.B.);; 3 Department of Computer Science (CAS), American University, Don Myers Technology & Innovation Building, 3501 Nebraska Avenue NW, Washington, DC 20016, USA; rcorizzo@american.edu

**Keywords:** cast iron, austempered ductile iron, YOLO, ResNet

## Abstract

The aim of this research is to develop and implement artificial intelligence models for the automatic detection of defects in the microstructures of austempered ductile iron (ADI). Our research used three different approaches, representing various categories of machine learning tasks: image classification (ResNet), pixel-wise segmentation (UNet), and object detection (YOLO). Each of the models were adapted to the specific characteristics of the dataset and tested on a collection of microstructural images prepared within the scope of the research. The data preparation process included clustering using the k-means method, morphological operations, generation of binary masks, conversion of labels into formats required by each architecture, and data augmentation to increase the diversity of training samples. The results demonstrated that ResNet achieved very high classification accuracy but did not provide spatial information about defect localization. UNet produced precise segmentation masks of martensitic regions, allowing for quantitative analysis of samples, although it required significantly higher computational resources and struggled with detecting very small defects. YOLO, in turn, enabled fast detection of defects in the form of bounding boxes. In summary, each model proved effective in a different context: ResNet for preliminary classification, UNet for detailed laboratory analysis, and YOLO for industrial detection tasks.

## 1. Introduction

Austempered Ductile Iron (ADI) is a special variety of ductile iron that, through appropriate heat treatment, achieves unique mechanical properties, combining high strength, impact strength, and wear resistance with good liquid metal castability and relatively low weight. The material’s microstructure is crucial for these properties, and its control is the subject of much research in materials science and production technology. Due to its properties, this material is widely used in sectors such as automotive, railways, defense, agriculture, and construction. The ADI microstructure is created by a two-stage heat treatment: austenitization at 820–950 °C and ausferritization at 250–400 °C for 0.5–4 h [1]. The result of these processes is a structure called ausferrite, consisting of austenite and ferrite in a “feathered” or “needle-shaped” form. The microstructure of ADI strongly depends on the casting wall thickness, which influences the cooling rate. Thinner walls (e.g., 2–3 mm) result in faster heat dissipation, leading to a greater number of fine graphite inclusions; thicker walls (e.g., 13 mm) result in fewer and larger graphite spheroids. Variable cooling conditions can also influence the presence of undesirable phases. One of the most important microstructural defects in ADI, particularly relevant to this work, is the presence of martensite. Martensite is not a desirable phase in the ADI structure. It can appear in areas where the transformation to ausferrite has not fully occurred—most often due to a mismatch in the aus tempering temperature or time, or due to too rapid a change in cooling conditions [2]. The presence of martensite negatively affects the material properties: it increases hardness at the expense of ductility, causes brittleness and susceptibility to cracking, and can be a source of microcrack initiation and damage propagation under fatigue conditions [3]. From a quality control perspective, rapid and accurate detection of martensite in the ADI microstructure is essential for eliminating defective components and ensuring repeatable production. Detecting defects in engineering materials is a key step in assessing their quality, strength, and suitability for industrial applications. Among the earliest methods used were non-destructive testing (NDT) techniques such as ultrasound, eddy current, magnetic particle testing, and radiography [4]. Artificial intelligence-based methods have also begun to be used to precisely identify defects [5,6,7]. Microscopic imaging, both optical and electronic (SEM, TEM), has been used in many industrial applications [8]. With the development of microscopy, the need for more efficient analysis of large amounts of image data arose. Initially, micrograph analysis was performed manually by experts. Although precise, this approach is characterized by being time-consuming, subjective, and having limited scalability [9]. Digital image processing methods have been intensively developed, enabling the automation of microstructure analysis. In the early period, classical algorithms for edge detection, segmentation, and mathematical morphology dominated [10]. They were used to classify microstructural phases, locate pores, cracks, or surface heterogeneities. Artificial neural networks (ANNs) began to outperform classical approaches in terms of accuracy and noise immunity [11] for various solutions [12]. Spiking neural networks with an improved pooling layer are also used [13]. Solutions based on convolutional neural networks (CNNs) offer high accuracy, operational speed, and the ability to scale analysis [14]. An approach that initially employs an autonomous recognition framework based on the Reinforced Open Set Algorithm (RAOS) and a feature subnetwork and strategy subnetwork with reinforcement learning and a deep reinforcement learning (DRL) model also yields good results. The created model undertakes a sequential decision-making task to optimize the feature subnetwork and strategy subnetwork [15]. Recent research has also emphasized the use of language models in data analysis [16]. Deep learning is used for the analysis of metal products [17], especially in the analysis of microstructures [18,19,20,21]. Basically, artificial intelligence in the context of microstructure analysis can perform three main functions: Classification—assigning an image to one of the classes (e.g., correct microstructure vs. defective) [22]; Segmentation—detecting and marking areas belonging to specific phases or defects (e.g., martensite) [23]; Object detection—locating and marking specific image elements (e.g., pores, microcracks, graphite particles) [24]. The UNet model has been used for precise segmentation of microstructural phases in high-strength steels, achieving high Dice coefficients (>0.9) [25]. The YOLO architecture, as a fast object detection network, finds applications in real-time production line analysis, including in the context of casting quality control [24]. ResNet, thanks to its deep structure and “residual connections” mechanism, is effective in classifying highly complex and low-contrast images, such as metallographic micrographs [26]. In the context of ADI, a particular challenge is the detection of martensite, which can take various forms and occur in small quantities. For this reason, AI models must be characterized not only by high sensitivity but also by robustness to interference resulting from image non-uniformity, polishing artifacts, and variability of heat treatment parameters. The literature also notes a growing interest in hybrid methods that combine classical image processing techniques (e.g., mathematical morphology, filtering) with AI algorithms, which increases the interpretability of models and facilitates their adaptation to various types of microstructures [14]. Based on literature analysis, it can be concluded that detecting defects in engineering materials, particularly ductile iron (ADI), is an issue of significant practical and research importance. The quality of this material’s microstructure largely determines its functional properties, therefore, effective identification of irregularities—such as martensite, porosity, or phase heterogeneity—is a crucial element of the quality control process. Traditional diagnostic techniques, based on non-destructive testing methods and light microscopy, allow for structural assessment but are limited by human factors, time-consuming, and difficult interpretation. In response to these limitations, artificial intelligence-based methods, particularly those utilizing convolutional neural networks, are gaining popularity.

A literature review shows that:

- AI enables the automation of microstructure analysis processes with high accuracy and repeatability,
- Convolutional neural networks such as ResNet, UNet, and YOLO are used in image classification, as well as in the segmentation and detection of microstructural objects,
- Previous research has focused primarily on general phase analysis, pore detection, discontinuities, and the classification of correct/incorrect structures, while the issue of precise automatic martensite detection is relatively rare and remains a research challenge.

This study focuses on microscopic images of materials such as ADI—particularly in the context of detecting subtle, difficult-to-detect structural defects. This work falls within this area. The aim of this study is to compare selected deep learning models (ResNet, UNet, YOLO) for their effectiveness in detecting martensite in the microstructure of ADI cast iron, which may contribute to improving quality control processes in industrial settings. At the same time, there are no effective methods that can be applied to collections of low numbers and limited diversity.

## 2. Materials and Methods

### 2.1. Methods

In recent years, we have observed a dynamic development of data analysis methods, in which Artificial Intelligence (AI) plays a central role. AI is becoming a tool that not only supports but increasingly replaces traditional information processing methods, especially where large datasets, subjective interpretation, or the need for high result repeatability are involved [27]. One of the key areas of AI is Machine Learning (ML), with the most advanced form of ML being deep learning based on the structure of artificial neural networks. Thanks to the layered architecture of deep networks, they can independently learn feature representations from raw data, making them particularly effective in image analysis [28,29]. One of the most important applications of AI in the context of this work is Image Processing, which encompasses a range of techniques enabling the analysis, transformation, segmentation, and classification of visual data. In the case of material microstructures, microscopic imaging is the primary source of information about the internal structure of the sample [30]. However, these images, despite their high quality, are complex, susceptible to noise, and often require advanced analytical tools to effectively extract features of interest, such as the presence of structural defects.

In tasks related to image analysis, and particularly in the recognition and localization of microstructural defects, Convolutional Neural Networks (CNNs) are currently the most effective and widely used approach. Their uniqueness stems from the fact that they can automatically learn feature representations from image data, eliminating the need for manual design of information extraction algorithms [31,32,33,34,35,36,37,38,39,40,41,42]. In this work, three popular architectures representing three approaches to image analysis were selected:

- ResNet (short for Residual Network) is one of the most influential deep neural network architectures, proposed [27]. This model significantly improved image classification performance in the ImageNet competition, and its main innovation was the introduction of so-called residual connections, which allow for training much deeper networks than previously possible [43]. In traditional deep neural networks, increasing the number of layers often led to the gradient vanishing problem—errors computed during backpropagation did not efficiently reach earlier layers, resulting in degraded training quality. ResNet addresses this problem by using residual blocks, which contain a direct connection (so-called skip connection) between the input and output of the block. This allows the model to learn the residual function F(x) = H(x) − x instead of the mapping H(x) directly, which significantly simplifies the optimization process [44]. Each residual block typically contains two or three convolutional layers and an addition operation that connects the original input with the output processed by the network layers. This structure allows for “shortcuts” in information flow, preserving the backscatter signal even with very deep architectures [44]. The original paper proposed several ResNet variants, differing in the number of layers: ResNet-18 and ResNet-34: networks with a shallow structure, with blocks containing two convolutional layers each; recommended for limited data or computational power. ResNet-50, ResNet-101, and ResNet-152: deeper versions that use so-called bottleneck blocks—three-layer blocks (1 × 1—3 × 3—1 × 1), allowing for a reduction in the number of parameters without sacrificing accuracy. In the context of this study, which aims to detect the presence of martensite in microscopic images, it was crucial to select a ResNet version that provides an appropriate compromise between depth, generalization ability, and computational cost. Using very deep variants, such as ResNet-101 or ResNet-152, could lead to overfitting due to the limited size and diversity of the available dataset [45]. On the other hand, too shallow networks (e.g., ResNet-18) may not be flexible enough to capture subtle structural differences between healthy microstructure and regions containing martensite. Therefore, we used ResNet50, an intermediate variant that provides a balance between architectural complexity and its generalization ability. This network contains 50 layers and uses classical residual blocks with two 3 × 3 convolutions, making it deep enough to detect complex patterns in image data, but not so complex that it requires a very large training set or hardware resources [45]. In the present study, ResNet was used to classify fragments of microscopic images as containing or not containing martensite. This architecture serves as a benchmark for more complex segmentation (UNet) and detection (YOLO) models, enabling comparison of results in terms of accuracy, precision, sensitivity, and inference time.
- UNet is a convolutional neural network architecture designed for precise semantic image segmentation, i.e., assigning a label to each input pixel. It was first presented in [23] as a tool for analyzing microscopic images in medicine [46]. Due to its versatility, efficiency, and ability to operate with limited datasets, UNet quickly found applications in many fields, including materials science [47]. The UNet architecture consists of two main parts: a contracting path and an expanding path. The contracting part operates analogously to classical convolutional networks—it consists of successive convolutional layers and pooling operations that reduce the image dimension while simultaneously increasing the network’s perceptual range. The goal of this part is to extract features with increasingly higher levels of abstraction. Then, in the expanding path, the image is successively restored to its original resolution using convolutional transposition operations (so-called upconvolution). At key points in the network, skip connections are used to copy feature maps from the corresponding levels of the shrinking path and connect them to the expansion layers. This allows UNet to combine global information (context) with local information (structure details), significantly improving segmentation quality, especially for fine, irregular structures [48]. UNet was designed for situations where small datasets are available, making it particularly attractive for materials science tasks, where labeled microscopic images are difficult and time-consuming to acquire [46]. The network can learn to effectively locate objects of interest even with a limited number of samples, as demonstrated in numerous publications on the segmentation of grains, pores, and non-metallic inclusions in metal microstructures [47]. In the context of this work, UNet was applied to segment areas containing martensite in microscopic images of ADI cast iron. This detection involves not only classifying the entire image but also pinpointing specific structural fragments where the defect is present. Thanks to its architecture, UNet is ideally suited for this task—it allows for high-precision marking of the contours and area occupied by the defect, even if its occurrence is irregular and localized. The advantage of using UNet is also the possibility of obtaining results in the form of segmentation masks, which can be subjected to further quantitative analysis—e.g., calculation of the area occupied by martensite, its shape, density or distribution relative to other phases. This type of data is extremely valuable in the context of quality control and prediction of mechanical properties of castings [47].
- YOLO (You Only Look Once) is a modern neural network architecture designed for fast object detection in images. Unlike classical approaches that analyze images in stages (e.g., region proposals, classification, localization), YOLO processes images end-to-end, performing detection and classification in a single pass through the network. This approach significantly increases performance and makes the model ideal for tasks requiring real-time operation, such as autonomous driving, monitoring, or industrial inspection [49].

The YOLO architecture divides the input image into a grid (e.g., 13 × 13 or 19 × 19 cells), and then predicts for each cell:

- The coordinates of a bounding box;
- The probability of object occurrence;
- The object class [50].

Unlike networks such as ResNet (whole image classification) or UNet (pixel-based segmentation), YOLO focuses on object localization and recognition by pinpointing the location of a fragment of interest and what it represents [50].

Since its inception in [43], the architecture has undergone intensive development. The most popular versions are:

- YOLOv1–v3: initial implementations, increasingly accurate and faster, but limited in localizing small objects.
- YOLOv4 (2020): significant improvement in accuracy thanks to the use of improved components such as CSPDarkNet53, PANet, and Spatial Pyramid Pooling.
- YOLOv5: not part of the official YOLO line, but one of the most commonly used in practice due to its availability, speed, and ease of implementation.
- YOLOv6–YOLOv8: versions based on newer network concepts and improved support for a variety of applications, including segmentation and classification in a single model [51].

Although YOLO was designed for real-time applications, its advantages make it equally useful in tasks where precise object localization within the context of the entire image is essential, not necessarily with maximum pixel detail. This is particularly important in microscopic image analysis, where full defect segmentation is not always necessary, and indicating their position and size is sufficient for further assessment [52].

In this work, YOLO (v7) was used to detect areas containing martensite in the structure of ADI cast iron. Thanks to its ability to rapidly process images and generate multiple location proposals, this model allows for the identification of microscopic fragments containing defects with good spatial precision. This is particularly important in the analysis of large samples, where manual review of entire images by an expert is time-consuming and prone to omissions.

Using YOLO in this context also allows for a comparison of its performance with approaches based on classification (ResNet) and segmentation (UNet), allowing for the assessment of which method is most effective depending on the application—for example, point detection vs. precise surface analysis.

Effective utilization of artificial intelligence methods for analyzing microscopic images requires appropriate preparation of the input data. The quality of the results obtained by deep learning models depends largely on how the images have been pre-processed. Image pre-processing includes a range of operations aimed at improving data quality, normalization, and the extraction of structural and textural information [45,46]. One of the methods used in the analysis of microstructure images is k-means clustering, as it allows for dividing the image into areas with similar characteristics. It can primarily be used to separate individual microstructure phases, such as austenite, ferrite, or martensite, based on differences in pixel intensity or textural properties. This enables the preparation of binary phase masks, which then serve as a starting point for further segmentation processes or quantitative analysis. K-means clustering is also applicable in identifying regions with distinct visual properties, allowing for the automatic isolation of areas of potential defects or material non-uniformities, which can be analyzed in subsequent steps using more advanced methods, such as neural networks [47,48]. Another group of techniques employed in microstructure image analysis are morphological operations (e.g., erosion, combinations of erosion and dilation, distance transforms, and boundary determination operations), which are particularly useful in the context of ADI microstructures for analyzing graphite grains and for cleaning segmentation masks obtained from neural network predictions, e.g., UNet [49]. The last important element of the data preparation process is feature extraction. Although deep learning models, such as ResNet or UNet, learn feature representations independently, utilizing classical extraction methods can support result analysis, improve error diagnosis, or serve as a comparative element [50].

This work utilized three different approaches to the analysis of ADI microstructures classification (ResNet), segmentation (UNet), and object detection (YOLO). Each of these approaches requires an appropriately chosen method of input data representation.

For classification models, such as ResNet, the most common way to prepare data is to assign appropriate class labels to the images. In this case, the input data are the images themselves, which are assigned one label, e.g., the presence or absence of a specific defect (martensite). This approach simplifies the task to binary classification, and the model learns to recognize general patterns indicating the occurrence of a defect. This approach has been widely described in the literature, where CNN models such as ResNet or VGG are trained on sets of images labeled zero or one to determine the presence or absence of a defect on the entire sample or a fragment thereof [51].

In the case of segmentation models, represented in this work by the UNet architecture, the preparation of binary masks is essential. Binary masks are images of the same resolution as the original input images, where each pixel is labeled with a value of 1 or 0. Pixels with a value of 1 indicate the presence of a defect or phase of interest, while pixels with a value of 0 represent the background or healthy material structure. This approach is commonly used in the literature, especially in the analysis of micrographs, where the detailed localization of defects is critical. The accuracy of the masks largely determines the effectiveness of the segmentation model [25].

For YOLO-type detection models, the input data require labels in the form of bounding boxes. Each defect is described by a set of coordinates defining a rectangle encompassing the defect area and the class of that object. This type of representation allows the model to efficiently learn the localization and classification of defects in images simultaneously. The application of this method is popular in industrial surface defect detection tasks, for example, on steel surfaces or castings, where fast indication of the defect location is important without the need for precise pixel segmentation [52].

It is worth noting that while all the mentioned models are based on convolutional neural networks, the differences in how the input data are prepared significantly impact the effectiveness of the solved problem. Each of these representations has its advantages and limitations. Binary classification is fast but does not specify the localization of defects; segmentation provides detailed spatial information at the cost of greater effort in labeling; while object detection partially combines the advantages of both approaches, allowing for quick and relatively precise localization of defects.

One of the key challenges in applying deep learning methods to the analysis of microscopic images is the limited number of available, labeled data. The goal of data augmentation is not only to increase the data size but also to improve model generalization and robustness to noise, scale variations, rotations, or illumination non-uniformity [53,54]. In the case of binary classification (e.g., ResNet), augmentation primarily serves to increase the diversity of examples with and without the defect. For segmentation (UNet) and detection (YOLO) networks, transformations must be applied in parallel to the images and their corresponding masks or detection frames to maintain semantic consistency [55]. The literature also emphasizes the importance of so-called targeted augmentation, which is adapted to the characteristics of the specific problem. In the context of metallic microstructures, this means, for example, avoiding excessive geometric deformations that could distort the actual phase morphology or incorrectly simulate defects. At the same time, it is suggested to apply augmentation in a controlled manner, considering the proportion of classes in the dataset and their representativeness [55]. In the research, data augmentation was used as one of the supplementary elements in the learning process of the ResNet, UNet, and YOLO models. This allowed for an increase in the effective number of training examples and improved the models’ ability to generalize to new, previously unobserved microscopic images.

### 2.2. Dataset

In the case of ADI microstructure analysis, the source data consists of microscopic images obtained from laboratory tests. For these to be effectively utilized by deep learning models, they must undergo a pre-processing stage, including normalization, segmentation, feature extraction, and the preparation of binary masks and detection frames. An additional supporting step is data augmentation, which allows for increasing the size and diversity of the training set.

The ADI microstructure is created through a two-stage heat treatment:

- Austenitizing at 820–950 °C;
- Isothermal hardening in the range of 250–400 °C for 0.5–4 h [1].

**Figure 1:**
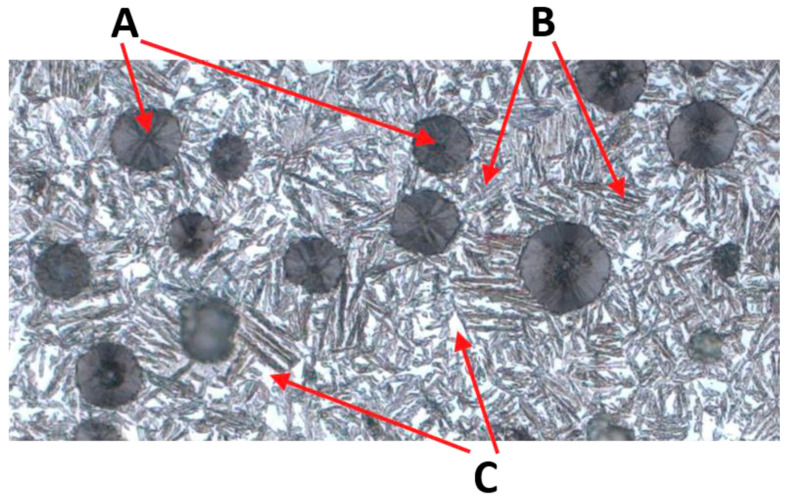
Example of microstructure of ADI cast iron, where: A—graphite, B—ferrite, C—austenite.

The result of these processes is a structure called ausferrite, consisting of austenite (Figure 1, light areas in the micrograph—C) and ferrite in a “feathered” or “acicular” form (Figure 1, darker areas with shades of gray, beige, and brown—B).

Depending on the hardening temperature, a distinction is made between:

- Upper ausferrite (T > 350 °C): higher austenite content, feathery ferrite;
- Lower ausferrite (T < 350 °C): dominance of acicular ferrite, smaller austenite areas [1].

**Figure 2:**
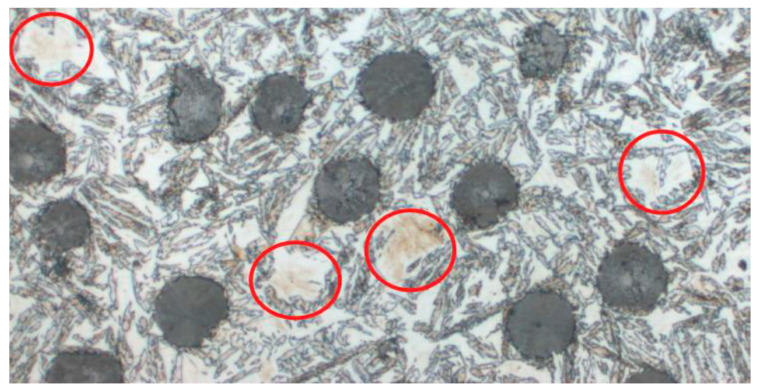
Microstructure with visible defects in the form of martensite.

The microstructure of ADI strongly depends on the casting wall thickness, which influences the cooling rate. Thinner walls (e.g., 2–3 mm) result in faster heat dissipation, leading to a greater number of fine graphite inclusions (Figure 1 A); in thicker walls (e.g., 13 mm), fewer and larger graphite nodules are observed. Variable cooling conditions can also influence the presence of undesirable phases. One of the most important microstructural defects in ADI, particularly relevant to this work, is the presence of martensite (Figure 2).

Martensite is not a desirable phase in the ADI structure. It can appear in areas where the transformation to ausferrite has not fully occurred—most often due to a mismatch in temperature or time of isothermal quenching, or due to too rapid a change in cooling conditions [2].

Under light microscopy, martensite has a characteristic, easily recognizable appearance:

- It appears as sharply pointed needles or diamond-shaped precipitates, often forming distinct zigzag structures.
- Color: red, ash-gray, navy blue, or almost black.
- Distribution: It can be dispersed or form clusters within former austenite regions [2].

The presence of martensite has a negative impact on the material’s properties:

- It increases hardness at the expense of ductility.
- It causes brittleness and susceptibility to cracking.

It can be a source of microcrack initiation and damage propagation under fatigue conditions [3]. From a quality control perspective, rapid and accurate detection of martensite in the ADI microstructure is crucial for eliminating defective components and ensuring production repeatability. Traditional micrograph analysis performed by materials science experts is time-consuming and prone to subjective errors. Therefore, the research focused on automating martensite detection using artificial intelligence methods, particularly deep learning models such as CNNs (ResNet, UNet, YOLO), which enable the recognition of microstructural phases based on microstructural elements. In the studied dataset (images of ADI microstructures), the presence of martensite was identified and confirmed based on characteristic image features. Detection of this phase is the main task of the considered convolutional neural network models, whose effectiveness will be compared in the presented studies.

The source material consisted of iron samples with varying wall thicknesses, subjected to a heat treatment process including austenitizing and austempering at different temperatures (100 samples). This made it possible to obtain a wide range of ausferritic structures, including samples containing martensite, which is the key defect analyzed in this work. Sample preparation included the process of polishing and etching the surface, in accordance with adopted metallographic procedures, to reveal the phase structures. Subsequently, images were recorded using an optical microscope (Zeiss, Oberkochen, Germany) at magnifications adjusted for the analysis of the ausferritic phase and microstructural defects. Image recording took place at established lighting parameters to ensure the comparability of the results obtained. The gathered research material included both samples with a structure typical for ADI (upper and lower ausferrite with graphite spheroids) and samples containing defects in the form of martensite and other undesirable elements, such as degraded forms of graphite or artifacts resulting from the preparation process of the polished surface. The presence of martensite was verified based on characteristic microscopic image features, such as needle-like or rhomboidal morphology and dark colors ranging from navy blue to black. The final dataset used in the experiments included both correct samples (defect-free) and samples with visible martensite. This set served as the basis for preparing input data in various formats, tailored to the requirements of the applied models: classification (ResNet), segmentation (UNet), and detection (YOLO).

In the context of ADI microstructures, a particular challenge is the presence of diverse elements, such as graphite spheroids, ferrite and austenite regions, and defects in the form of martensite. These elements can have similar intensity and texture, making their automatic differentiation difficult. Therefore, the use of appropriate pre-processing techniques allows for the extraction of important image features, which are then utilized by classification, segmentation, and detection models in subsequent stages. The most commonly used steps in data pre-processing include morphological operations, image segmentation and clustering, as well as feature extraction and scale normalization [56,57,58].

The first stage of data preparation involved dividing large, microscopic images into four fragments. This achieved several effects simultaneously: the size of a single sample was reduced, facilitating further calculations, the number of input data for the models was increased, and convolutional networks were enabled to better capture local features characteristic of the phases under study.

**Figure 3:**
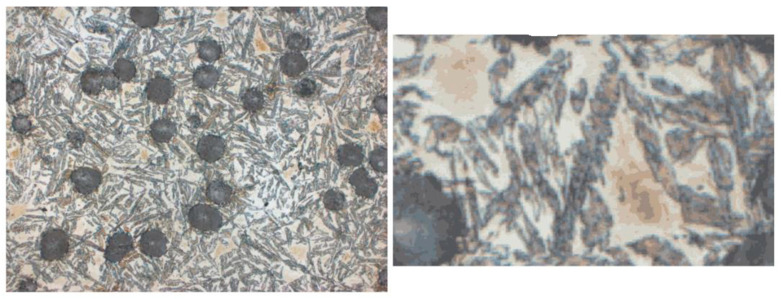
Clustering results for k = 14—original image and clustered, approximated image.

Subsequently, k-means (Figure 3) clustering was applied to the image fragments prepared in this way. The algorithm was implemented in Python using the scikit-learn library, and the result was binary masks saved in the output directory. After obtaining binary masks using k-means clustering, additional cleaning and standardization were necessary. This is because the clustering process, although effective in differentiating individual material phases, also generates minor distortions: isolated pixels, discontinuities within areas, or small empty spaces inside structures. To prepare the data for further analysis and model training, a set of morphological operations was applied, implemented in Python (v3.12) using the OpenCV library (v4.10.0).

**Figure 4:**
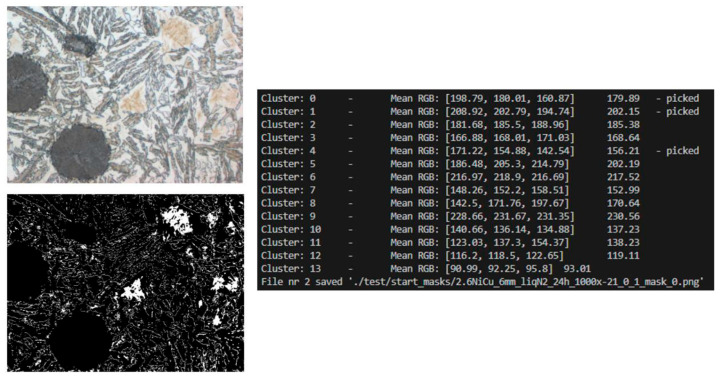
Input image, output code message, and output binary mask.

**Figure 5:**
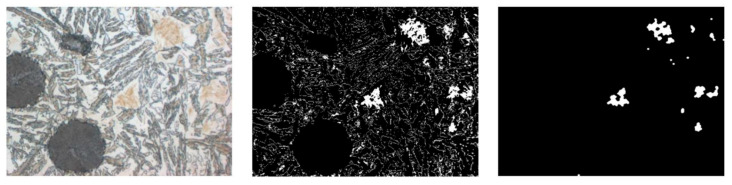
Summary of the results of morphological operations. Original image, binary mask, mask after morphological operations.

The final stage of data preparation before the model learning process was the augmentation of images and their corresponding binary masks 9, (Figure 4). The augmentation was implemented in Python using the Albumentations library, which offers a wide range of image transformations and, importantly, ensures their simultaneous application to both input images and their corresponding masks. The following transformations were applied: mirror reflections (horizontal and vertical), transposition, random rotations by a multiple of 90°, and Grid Distortion. Thanks to the conducted process, the size of the dataset was significantly increased, which was particularly important when training the UNet and YOLO models. Segmentation and detection networks are sensitive to the diversity of input data, and the limited number of original images could lead to the problem of overfitting. Augmentation allowed for the artificial introduction of image variants with different orientations and local deformations, which translated into greater robustness of the models to data non-uniformity. It is worth noting that the selection of specific augmentation methods and parameters was preceded by a series of experiments. Different combinations of transformations were tested to find a balance between increasing data diversity and maintaining the structural realism of microstructure images and their binary masks. The final set of transformations (Figure 5) ensured both the increase in the dataset and consistency with the actual morphology of martensite.

### 2.3. Experiments

After collecting the research material and completing the input data preparation process, the actual experimental part began. Its goal was to implement and compare selected neural network architectures in the context of detecting martensite in ADI microstructures. The applied models represented three different approaches to the problem:

- ResNet—image classification (information about the presence or absence of a defect),
- UNet—semantic segmentation (precise delineation of defect areas),
- YOLO—object detection (localization of defects in the form of bounding boxes).

The research process included several stages: preparation of training, validation, and test sets, implementation of the models in the Python environment using machine learning libraries, and then training the networks with specified hyperparameters. The results of each model were evaluated based on a set of quality metrics adapted to the nature of the task: accuracy, precision, recall, F1-score, IoU, Dice, and mAP.

#### Experiment Preparation

Before starting the actual model training, it was necessary to prepare the data in a format tailored to the nature of the respective research tasks. After the augmentation stage, additional operations were performed to obtain a consistent and organized set that could be used by classification, segmentation, and detection networks.

The first step was the division of images into fragments of 256 × 256 pixels. This size was chosen based on the literature, which indicates that smaller image fragments allow convolutional networks to better recognize local features characteristic of individual microstructure phases. The division of large micrographs into regular fragments also had a practical dimension: it increased the size of the set, which reduced the risk of model overfitting, and simultaneously allowed for effective use of GPU memory during training. An additional advantage of this operation was the removal of the ‘edges’ of the original images, which contained noise and lower sharpness.

Next, the images were labeled for the occurrence of defects. To do this, for each fragment, it was checked whether an area corresponding to martensite was present in its corresponding binary mask. If pixels marked as a defect were present in the mask, the entire fragment received the label “1” (defect present). Otherwise, the fragment was labeled “0” (no defect). This resulted in a dataset adapted for training the ResNet classifier, where each image was assigned a binary label.

The next step was to separate the training, validation, and test sets. This process was simplified by the prior labeling of images as containing or not containing defects. The division was performed according to the adopted convention, where:

- The training set included the majority of samples and was used to train the models;
- The validation set served as the basis for selecting hyperparameters and monitoring the learning process;
- The test set contained previously unused data and allowed for the final evaluation of model quality.

All data was organized according to an established file and directory naming convention. Uniform naming schemes were used for images and masks, making it possible to automatically pair images with their corresponding masks. Additionally, each part of the set (training, validation, test) was placed in a separate directory, which significantly facilitated the subsequent process of learning and testing the models. The data prepared in this way constituted a complete and organized input set, ready for use in experiments with three different neural network architectures: ResNet, UNet, and YOLO.

## 3. Results

**Figure 6:**
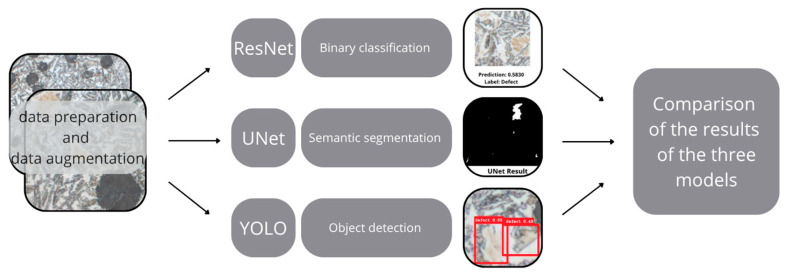
The general diagram of the developed solution.

The general diagram of the developed solution is shown in Figure 6.

### 3.1. ResNet Model Results

The ResNet50 architecture, one of the popular variants of residual networks, was used for the task of classifying microstructure fragments based on the presence of defects in the form of martensite. The model was adapted for binary classification by modifying the output layer to return two classes: “0”—no defect, and “1”—defect present. Alternatively, a deeper variant, ResNet152, was also considered, but due to computational complexity and training time, ResNet50 was ultimately chosen, proving to be a compromise between accuracy and efficiency.

Input images were transformed to a resolution of 256 × 256 pixels, normalized, and converted to a tensor. In the learning process, the CrossEntropyLoss function, which is standard in multi-class classification, and the Adam optimizer, known for effectively combining the advantages of adaptive methods and classical gradient approaches, were used. The learning process was conducted for 20 epochs with a batch size of 16 and an initial learning rate of 10^−3^. Mixed precision computations were also used, which allowed for faster training while maintaining numerical stability. After each epoch, the model state was saved in a checkpoint file, enabling the reproduction of results and further experiments.

**Figure 7:**
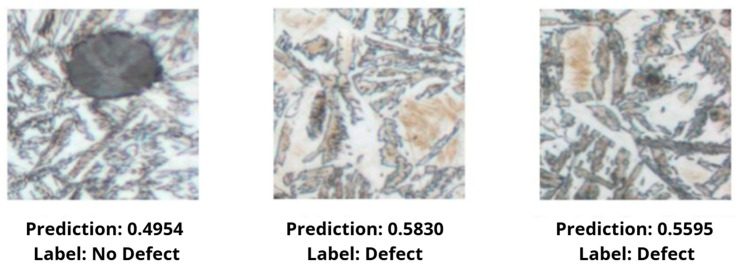
Comparison of selected model results.

After the network training was completed, data from the test set was introduced into the model, and the results were observed. Selected results are presented in Figure 7.

**Figure 8:**
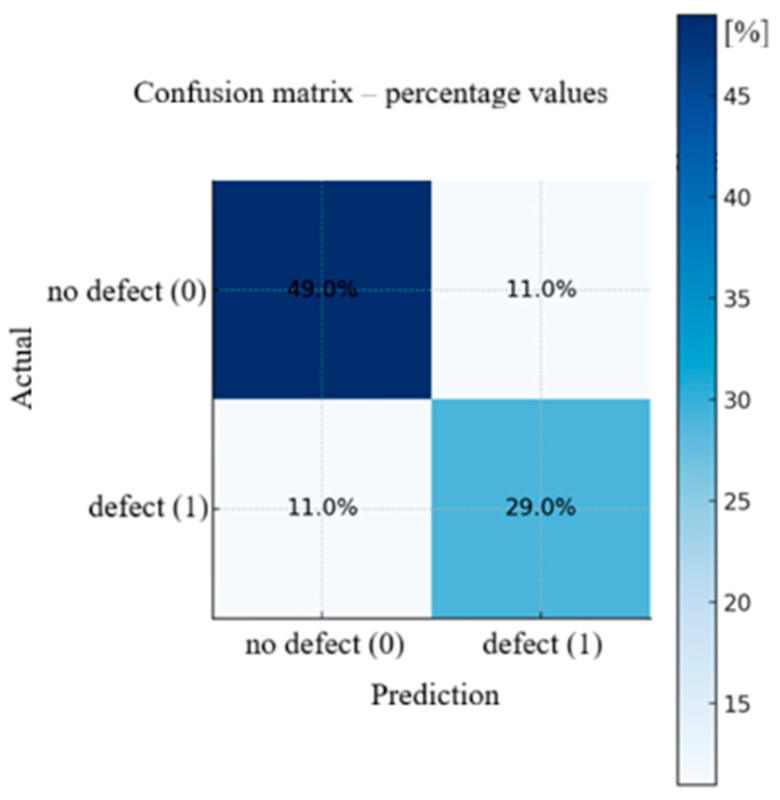
Table of errors in defect presence prediction using the ResNet model.

The obtained results indicate that ResNet50 effectively distinguishes fragments containing defects from those without them. Analysis of the confusion matrix (Figure 8) showed that the model performs very well in the case of clear clusters of martensite, while difficulties arise in images where the defect occurs as small or blurred structures. The model correctly classified 78% of cases, of which 49% were correct recognitions of no defect, and 29% were correct detections of a defect. Errors occur in 11% of cases for both classes. This means that some fragments where the boundary between martensite and the background was indistinct led to incorrect model decisions.

In summary, ResNet50 is an effective tool for rapid classification of microstructure images, providing simple information about the presence or absence of a defect. Its advantage is high effectiveness and moderate computational requirements, which makes it useful in preliminary analysis tasks. However, the lack of precise defect localization in the image remains a limitation, justifying the application of more complex architectures, such as UNet or YOLO, in the later part of the work.

### 3.2. UNet Model Results

The UNet architecture with a single output channel (out_channels = 1) was used for the martensite area segmentation task, which corresponds to binary segmentation (defect/background). Input images were scaled to 256 × 256 pixels and normalized, then converted to tensors. Segmentation masks were loaded as binary images and converted to a floating-point type and channel dimension expanded (targets.float().unsqueeze(1)) during the learning phase so that their shape matched the network output. The loss function used was BCEWithLogitsLoss, meaning the network returned raw logits (without the sigmoid function on the last layer), and thresholding to a binary form occurred only at the evaluation stage (typically at a threshold of 0.5). Adam with a learning rate of 10^−4^, a batch size of 8, and a number of epochs equal to 100 was used for optimization.

**Figure 9:**
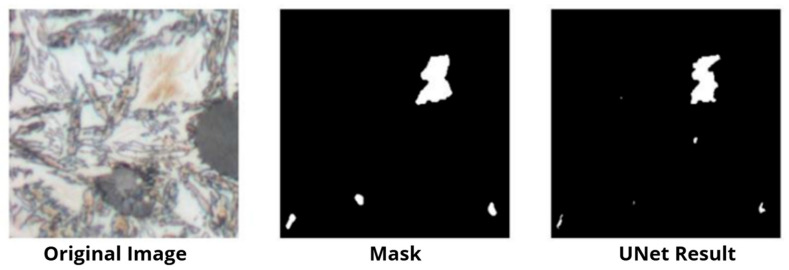
One of the results of tests with the UNet model test dataset.

Part of the data was allocated for training and part for validation; image-mask pairing was ensured by dedicated loaders (get_loaders), which applied identical geometric transformations (scaling and normalization) to both sets. Commented-out random transformations (rotations, reflections) were left in the code, which were used in the earlier stage of augmenting the entire set; in this specific script, a deterministic validation stage was prioritized. During training, after each epoch, the model checkpoint (weights and optimizer state) was saved, quality metrics were calculated on the validation set (check_accuracy), and example predictions were generated in the form of output images (save_predictions_as_imgs)—this allowed for ongoing control of both numerical values (e.g., IoU, Dice) and the visual quality of the masks. After the network training was completed, data from the test set was introduced into the model, and the results were observed. A selected result is presented in Figure 9.

After 22 epochs of learning, lasting a total of approximately 19 h, results confirming the effectiveness of martensite segmentation were obtained. Quality indicators for the defect class reached values of IoU ≈ 0.66 and Dice ≈ 0.81, which well reflects the model’s ability to precisely capture larger, compact areas of martensite. Difficulties, however, arose in the case of very fine, scattered islands, which were more often overlooked (false negatives) or oversegmented as fragments of defects (false positives). The obtained results confirmed the usefulness of UNet for precise, pixel-level martensite segmentation: the values of the IoU and Dice coefficients remained at a high level for samples where the defect formed clear, continuous areas. Weaker results were observed in cases with very fine, scattered structures or at low-contrast phase boundaries—where the model was susceptible to oversegmentation or underestimation of the defect area. A review of the saved visual results indicates that the inclusion of prior mask cleaning (morphological operations) and consistent data normalization positively influence the stability of learning and the shape of predicted contours. From a practical point of view, UNet provides not only a decision about the presence of a defect but also its location and shape, enabling further quantitative inference (e.g., the area fraction of martensite) and complementing the ResNet classifier. The main drawback of this solution is the erroneous indication of an area resembling martensite.

### 3.3. YOLO Model Results

The YOLO (You Only Look Once) architecture, one of the most frequently used neural networks in object localization tasks, was applied for the purpose of defect detection in the form of martensite. This model, unlike ResNet and UNet, does not return binary information or a pixel mask but localizes defects in the form of rectangular bounding boxes along with an assigned class and detection probability. This approach allows for rapid identification of microstructure fragments requiring further analysis.

To prepare the input data, binary masks obtained in earlier stages were utilized. For each image, rectangles surrounding coherent areas of martensite were determined, and then converted to the YOLO format, including the coordinates of the box center, its width and height, and the class label. This process was fully automated and did not require manual labeling.

**Figure 10:**
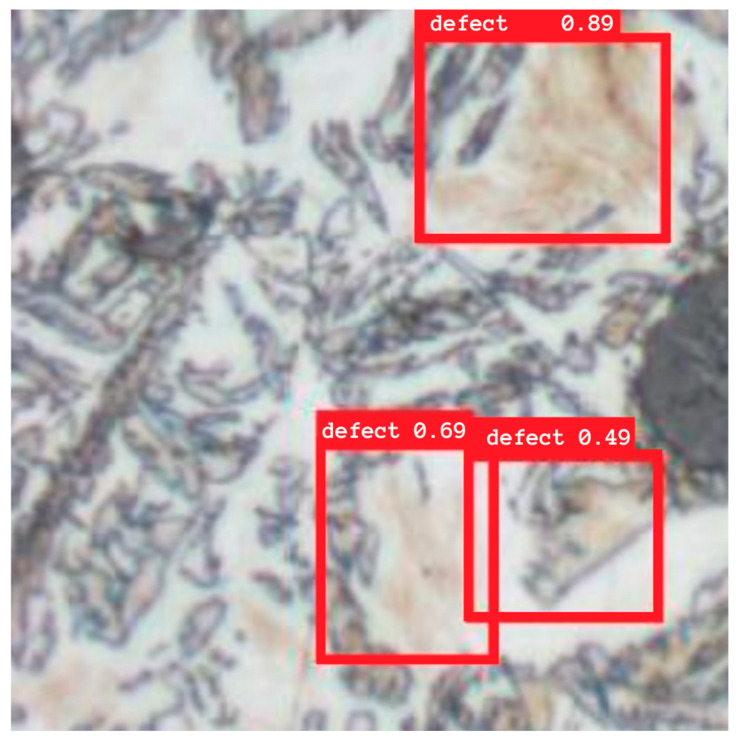
Selected result of the trained YOLOv8 model.

On the basis of the prepared data, the YOLOv8 network was trained, configured for a single-output problem. The network was trained from scratch, without the use of pre-trained weights, as images of material microstructures do not appear in public datasets. In the configuration, an input image resolution of 256 × 256 pixels, a batch size of 32, a set number of epochs at 80, and a learning rate of 10^−3^ were adopted. The Adam algorithm was used for optimization, and the learning process was conducted on a graphics card utilizing parallel computing. Figure 10 presents an example result of four hours of training the YOLO network model. 

Quality assessment was performed based on standard detection metrics: precision, recall, and mean Average Precision (mAP) calculated at an IoU = 0.5 threshold. The results indicate that YOLO effectively localizes larger clusters of martensite, achieving a high precision value and a relatively small number of false alarms. The model encountered the greatest difficulties in the case of very fine or scattered defects, which were sometimes underestimated or completely missed. Additional problems included areas of brownish ferrite resembling martensite. A comparison of the YOLO detection boxes with the segmentation masks obtained using UNet confirmed this observation YOLO allows for quick detection of defect locations but with less accuracy in reproducing their shape.

In summary, YOLO proved particularly useful in applications requiring a quick response, such as in preliminary quality control systems. Compared to ResNet and UNet, it provides a compromise between operating time and localization accuracy. Although it does not provide as detailed information as segmentation, it is a valuable complement to the previous approaches and allows for the rapid identification of areas that can then be subjected to more detailed analysis.

### 3.4. Comparison of Model Results

In this work, three approaches to defect detection in microstructures were applied: image classification of fragments using ResNet, pixel-level segmentation using UNet, and object detection using YOLO. Each model differs in both its mechanism of action and the nature of its results, which translates into their usefulness in different research and practical contexts. Table 1 summarizes the obtained results and their interpretation.

Analyzing the results, it can be observed that ResNet is the most computationally efficient solution and performs very well in quickly detecting whether a given fragment contains a defect or not. This makes it a tool for preliminary classification of large image sets, but its biggest limitation is the lack of spatial information. In practice, this means that the model can indicate the presence of martensite but will not show its distribution or size.

In turn, UNet allows for pixel-level segmentation, which provides the most accurate image of the defect: its shape, area, and location. This is extremely important in laboratory analyses, as it enables the quantitative estimation of the martensite fraction in the sample. High IoU and Dice values indicate a high consistency between the predicted and actual masks. The disadvantage of UNet is the longer training and prediction time, as well as difficulties in detecting very fine or blurred structures, which are sometimes treated as noise.

The YOLO model represents a compromise between the two methods above. It operates significantly faster than UNet, while providing more information than ResNet, as it indicates the approximate location of the defect in the form of a bounding box. The mAP values obtained show that YOLO handles larger clusters of martensite well, but struggles with correctly capturing small, irregular islands. Its advantage is the very fast prediction time, making it a suitable candidate for applications in quality control systems in near-real-time.

## 4. Discussion

Comparing the models, it can be observed that each fulfills a different role:

- ResNet is best suited for the quick classification of large image sets and filtering out defect-free fragments.
- UNet is effective in precise laboratory analysis, where accurate information about the defect’s shape and size is important.
- YOLO provides high-time efficiency and reliable defect localization, making it attractive for industrial applications.

From an engineering practice and materials research perspective, it can therefore be concluded that a hybrid approach yields the best results: ResNet as a preliminary filter, YOLO for quick defect localization, and UNet for detailed analysis of its shape and size. Such a combination of methods could form the basis for a comprehensive system for automated microstructure assessment.

The results obtained from the conducted experiments indicate the high effectiveness of the applied methods in the task of detecting defects in ADI microstructures, with each of the utilized architectures playing a different role and being characterized by different limitations. It is worthwhile to compare these with the results presented in the literature to better situate the conducted research in a broader scientific context.

In the case of image classification using ResNet, the observations of authors of earlier works were confirmed, indicating that residual networks perform well with images of materials with complex texture [e.g., applications in weld analysis or detection of surface defects in metals]. The high accuracy obtained in the experiment is consistent with reported values in the literature, although it should be emphasized that ResNet does not allow for indicating the defect location, which may be insufficient in many industrial applications. Thus, ResNet is primarily effective as a tool for preliminary classification or filtering of large image sets.

The application of UNet for binary segmentation of martensite allowed for obtaining qualitative and quantitative results comparable to those presented by other researchers in microstructure segmentation tasks. The literature emphasizes that UNet is one of the most frequently chosen architectures for material analysis, partly due to its ability to accurately reproduce object contours. This feature was also confirmed in this work IoU and Dice values reached high levels, and visual comparison of the masks indicates a high consistency with the reference data. Difficulties arose, however, in the case of very fine or scattered defects, which is consistent with the reports of other authors who point to UNet’s problems in capturing objects with a small area and low contrast.

The YOLO architecture, although less frequently used in strictly materials literature than in medical image analysis or industrial vision systems, also confirmed its usefulness in the defect detection task. Similar to other publications, YOLO proved to be very fast and effective in detecting larger objects but had a tendency to miss fine structures. The mAP results obtained in the experiment fall within the range reported in the literature for tasks of similar complexity, although it should be noted that in this work, the network was trained from scratch without using pre-trained weights, which may have influenced the final accuracy.

The comparison of the three approaches shows that there is no single universal method that best solves the problem of martensite detection. Each model proves effective in a different context: ResNet—in quick classification, UNet—in precise laboratory analysis, and YOLO—in applications requiring immediate defect detection. Such conclusions are consistent with the trend visible in the literature, where the need to combine different methods and create hybrid systems is increasingly emphasized.

Ultimately, it can be stated that the presented research confirms the current state of knowledge regarding the use of deep learning methods for material microstructure analysis, and simultaneously indicates directions for further development primarily in the area of increasing the accuracy of detecting fine defects and incorporating newer neural network architectures, such as models based on attention mechanisms (vision transformers).

## 5. Conclusions

The most important result of the work was the development and implementation of three distinct approaches to defect detection in ADI ductile iron microstructures: image classification (ResNet), pixel-level segmentation (UNet), and object detection (YOLO). The comparison of the results obtained by the individual models allowed for a comprehensive assessment of their usefulness in martensite analysis, indicating both their strengths and limitations. Key achievements include:

- The preparation of an original dataset including microstructure images along with their corresponding binary masks and detection labels. This process involved division of large images into smaller fragments, application of k-means clustering, morphological operations, and data augmentation, which ensured an adequate number and diversity of samples;
- The implementation of three neural network architectures adapted to the specifics of the task: ResNet as a binary classifier, UNet as a segmentator, and YOLO for the detection of rectangular bounding boxes;
- Conducting the full process of learning and validation using the Python environment and deep learning libraries, including hyperparameter selection, saving model checkpoints, and monitoring result quality in successive epochs;
- The qualitative and quantitative analysis of the obtained results based on a set of metrics (accuracy, precision, recall, F1-score, IoU, Dice, mAP), which allowed for a reliable comparison of the effectiveness of the individual approaches;
- The identification of potential practical applications: ResNet as a fast preliminary classifier, UNet as a tool for detailed laboratory analysis, and YOLO as a method for rapid detection in the context of industrial control.

The realized research confirmed the effectiveness of deep neural networks in the analysis of material microstructures and represents a step towards automating the defect detection process, which until now required significant involvement of specialists and subjective microscopic evaluation.

The main limitation was the size and nature of the dataset. ADI microstructure images were obtained under laboratory conditions, and although the dataset was supplemented with an augmentation process, its size remains small in comparison to popular datasets used in deep learning. This limits the possibility of fully utilizing the potential of large models, such as ResNet152 or more complex YOLO variants. Additionally, the lack of publicly available datasets with similar characteristics prevents the use of fully effective transfer learning. A second limitation is the difficulty in detecting very small and irregular defects. UNet, despite high-quality segmentation, had a tendency to miss fine martensite islands, and YOLO tended to treat them as noise and not include them in the detection boxes. This is a problem commonly described in the literature, resulting from both architectural limitations and insufficient representation of such cases in the dataset. Another limiting factor is the time-consuming nature of the data preparation process. Although the process was fully automated, it includes several stages (k-means clustering, morphological operations, mask creation, YOLO label generation), which in the case of large industrial datasets may require further optimization and simplification.

In conclusion, although the presented approaches provided promising results, further research development towards expanding datasets, implementing newer models, and integrating methods has the potential to significantly increase the effectiveness and usefulness of automatic microstructure analysis systems in industrial and scientific practice.

## Figures and Tables

**Table 1:** Comparison of the results of the three models.

Model	Task	Metrics	Time	Advantages	Limitations
ResNet50	Recognizing the presence of a defect in the image	Accuracy: 78% Precision: 72.5% Recall: 72.5% F1-score: 0.725	20 epochs (~13 h)	Speed of classification, simpler training data preparation	Lack of information about defect location and shape; predictions less certain (0.4–0.6)
UNet	Pixel-by-pixel segmentation of martensite areas	IoU: 0.66 Dice: 0.81	22 epochs (~19 h)	Very good segmentation precision for larger areas, detailed shape reproduction	Difficulties with fine, scattered islands; greater time requirements
YOLOv8	Defect localization via bounding boxes	mAP: 0.83 Precision: 0.86 Recall: 0.74 F1-score: 0.79	80 epochs (~3 h)	Very fast prediction (0.02–0.03 s/image), effective detection of larger defects	Worse detection of the smallest structures, boxes do not accurately reflect shapes

## Data Availability

The data that support the findings of this study are available on request from the corresponding author, [D.W.-K. and S.G.]. The data are not publicly available due to the database requires a lot of financial resources and is the subject of an as yet unpublished doctoral dissertation.

## References

[1] Krawiec H., Lelito J., Mróz M., Radoń M. (2023). Influence of Heat Treatment Parameters of Austempered Ductile Iron on the Microstructure, Corrosion and Tribological Properties. Materials.

[2] Polishetty A., Pan B., Pasang T., Littlefair G. Microstructural Study on Strain Induced Transformation in Austempered Ductile Iron Using Heat Tinting. Proceedings of the ASME 2010 International Manufacturing Science and Engineering Conference.

[3] Górny M., Tyrała E. (2013). Effect of cooling rate on microstructure and mechanical properties of thin-walled ductile iron castings. J. Mater. Eng. Perform..

[4] Silva M.I., Malitckii E., Santos T.G., Vilaça P. (2023). Review of conventional and advanced non-destructive testing techniques for detection and characterization of small-scale defects. Prog. Mater. Sci..

[5] Jia Y., Chen G., Zhao L. (2024). Defect detection of photovoltaic modules based on improved Varifocal Net. Sci. Rep..

[6] Huang Z., Zhang C., Ge L., Chen Z., Lu K., Wu C. (2024). Joining Spatial Deformable Convolution and a Dense Feature Pyramid for Surface Defect Detection. IEEE Trans. Instrum. Meas..

[7] Shao R., Zhou M., Li M., Han D., Li G. (2024). TD-Net:tiny defect detection network for industrial products. Complex Intell. Syst..

[8] Dehaerne E., Dey B., Blanco V., Davis J. (2025). Scanning electron microscopy-based automatic defect inspection for semiconductor manufacturing: A systematic review. J. Micro/Nanopatterning Mater. Metrol..

[9] AI Image Evaluation vs. Manual Microstructure Analysis—Advantages and Disadvantages. https://mivia.ai/en/2024/01/ki-bildauswertung-vs-manuelle-mikrostrukturanalyse-vor-und-nachteile/.

[10] Campbell A., Murray P., Yakushina E., Marshall S., Ion W. (2018). New methods for automatic quantification of microstructural features using digital image processing. Mater. Des..

[11] Bai J., Wu D., Shelley T., Schubel P., Twine D., Russell J., Zeng X., Zhang J. (2025). A Comprehensive Survey on Machine Learning Driven Material Defect Detection. ACM Comput. Surv..

[12] Liu Q., Liu M., Wang C., Wu Q.M.J. (2024). An efficient CNN-based detector for photovoltaic module cells defect detection in electroluminescence images. Sol. Energy.

[13] Wang Z., Li S., Xuan J., Shi T. (2025). Biologically inspired compound defect detection using a spiking neural network with continuous time–frequency gradients. Adv. Eng. Inform..

[14] Ferguson M., Ak R., Lee Y.-T.T., Law K.H. (2018). Detection and Segmentation of Manufacturing Defects with Convolutional Neural Networks and Transfer Learning. Smart Sustain. Manuf. Syst..

[15] Wang Z., Xuan J., Shi T. (2024). An autonomous recognition framework based on reinforced adversarial open set algorithm for compound fault of mechanical equipment. Mech. Syst. Signal Process..

[16] Peng C., Peng J., Wang Z., Wang Z., Chen J., Xuan J., Shi T. (2025). Adaptive fault diagnosis of railway vehicle on-board controller with large language models. Appl. Soft Comput..

[17] Nowroth C., Gu T., Grajczak J., Nothdurft S., Twiefel J., Hermsdorf J., Kaierle S., Wallaschek J. (2022). Deep Learning-Based Weld Contour and Defect Detection from Micrographs of Laser Beam Welded Semi-Finished Products. Appl. Sci..

[18] Zhao J., Shen C., Huang M., Qi Y., Chai Y., Zheng S. (2025). Deep learning accelerated micrograph-based porosity defect quantification in additively manufactured steels for uncovering a generic process-defect-properties relation. Mater. Charact..

[19] Nithin A.M., Perumal M., Davidson M.J., Srinivas M., Rao C.S.P., Harikrishna K., Jagtap J., Bhowmik A., Santhosh A.J. (2025). Segmentation Studies on Al-Si-Mg Metallographic Images Using Various Different Deep Learning Algorithms and Loss Functions. Eng. Rep..

[20] Wang Y., Wei X., Wang C., Xu W. (2025). Physical Metallurgy-Guided Machine Learning for Strength-Plasticity Optimization in Aluminum Alloys. Mater. Today Commun..

[21] Müller M., Stiefel M., Bachmann B.-I., Britz D., Mücklich F. (2024). Overview: Machine Learning for Segmentation and Classification of Complex Steel Microstructures. Metals.

[22] DeCost B.L., Lei B., Francis T., Holm E.A. (2019). High Throughput Quantitative Metallography for Complex Microstructures Using Deep Learning: A Case Study in Ultrahigh Carbon Steel. Microsc. Microanal..

[23] Ronneberger O., Fischer P., Brox T. U-Net: Convolutional Networks for Biomedical Image Segmentation. Lecture Notes in Computer Science (including subseries Lecture Notes in Artificial Intelligence and Lecture Notes in Bioinformatics). Proceedings of the Medical Image Computing and Computer-Assisted Intervention.

[24] Liu Y., Liu Y., Guo X., Ling X., Geng Q. (2025). Metal surface defect detection using SLF-YOLO enhanced YOLOv8 model. Sci. Rep..

[25] Biswas M., Pramanik R., Sen S., Sinitca A., Kaplun D., Sarkar R. (2023). Microstructural segmentation using a union of attention guided U-Net models with different color transformed images. Sci. Rep..

[26] He K., Zhang X., Ren S., Sun J. Deep residual learning for image recognition. Proceedings of the IEEE Computer Society Conference on Computer Vision and Pattern Recognition.

[27] The Impact of AI on the Engineering Field|JHU EP. https://ep.jhu.edu/news/the-impact-of-ai-on-the-engineering-field/.

[28] What Is Machine Learning? Definition, Types, Tools & More|DataCamp. https://www.datacamp.com/blog/what-is-machine-learning.

[29] An Overview of Deep Learning for Image Processing—Christian Garbin’s Personal Blog. https://cgarbin.github.io/deep-learning-for-image-processing-overview/.

[30] Ge M., Su F., Zhao Z., Su D. (2020). Deep learning analysis on microscopic imaging in materials science. Mater Today Nano.

[31] Tu S., Vo P. (2024). Microstructural Feature Extraction by a Convolutional Neural Network for Cold Spray of Aluminum Alloys. J. Therm. Spray Technol..

[32] Gea M.N., Wanayumini W., Rosnelly R. (2025). Impact of Hyperparameter Tuning on CNN-Based Algorithm for MRI Brain Tumor Classification. J. Tek. Inform..

[33] Han Y., Li R., Yang S., Chen Q., Wang B., Liu Y. (2022). Center-environment feature models for materials image segmentation based on machine learning. Sci. Rep..

[34] Wankhade N., Sale V., Yadav R., Jikar P., Gadgekar S., Dhokey N. (2024). Metallurgical microstructure classification using CNN: A comprehensive study on heat treatment analysis for steel. Mater. Today Proc..

[35] Convolutional Neural Networks: A Complete Guide|Medium. https://medium.com/@alejandro.itoaramendia/convolutional-neural-networks-cnns-a-complete-guide-a803534a1930.

[36] Convolutional Neural Networks (CNNs) and Layer Types—PyImageSearch. https://pyimagesearch.com/2021/05/14/convolutional-neural-networks-cnns-and-layer-types/.

[37] Pehlivanoğlu M.K., İnCe I., Kından B.A., Eker A.G., Doğan İ. (2025). Towards advanced brain tumor segmentation: A novel hybrid architecture integrating UNet, FCN, and YOLO models on the newly introduced BTS-DS 2024 dataset. Eur. Phys. J. Spec. Top..

[38] Saxena A. (2022). An Introduction to Convolutional Neural Networks. Int. J. Res. Appl. Sci. Eng. Technol..

[39] Choudhary K., DeCost B., Chen C., Jain A., Tavazza F., Cohn R., Park C.W., Choudhary A., Agrawal A., Billinge S.J.L. (2022). Recent advances and applications of deep learning methods in materials science. NPJ Comput. Mater..

[40] He K., Zhang X., Ren S., Sun J. Identity mappings in deep residual networks. Lecture Notes in Computer Science (including subseries Lecture Notes in Artificial Intelligence and Lecture Notes in Bioinformatics). Proceedings of the Computer Vision—ECCV 2016.

[41] Gebhardt C., Trimborn T., Weber F., Bezold A., Broeckmann C., Herty M. (2020). Simplified ResNet approach for data driven prediction of microstructure-fatigue relationship. Mech. Mater..

[42] Medghalchi S., Kortmann J., Lee S.-H., Karimi E., Kerzel U., Korte-Kerzel S. (2024). Automated segmentation of large image datasets using artificial intelligence for microstructure characterisation and damage analysis. Mater. Des..

[43] Redmon J., Divvala S., Girshick R., Farhadi A. You Only Look Once: Unified, Real-Time Object Detection. Proceedings of the 2016 IEEE Conference on Computer Vision and Pattern Recognition (CVPR).

[44] Terven J., Córdova-Esparza D.-M., Romero-González J.-A. (2023). A Comprehensive Review of YOLO Architectures in Computer Vision: From YOLOv1 to YOLOv8 and YOLO-NAS. Mach. Learn. Knowl. Extr..

[45] Salvi M., Acharya U.R., Molinari F., Meiburger K.M. (2021). The impact of pre- and post-image processing techniques on deep learning frameworks: A comprehensive review for digital pathology image analysis. Comput. Biol. Med..

[46] Luengo J., Moreno R., Sevillano I., Charte D., Peláez-Vegas A., Fernández-Moreno M., Mesejo P., Herrera F. (2022). A tutorial on the segmentation of metallographic images: Taxonomy, new MetalDAM dataset, deep learning-based ensemble model, experimental analysis and challenges. Inf. Fusion.

[47] Dhanachandra N., Manglem K., Chanu Y.J. (2015). Image Segmentation Using K -means Clustering Algorithm and Subtractive Clustering Algorithm. Procedia Comput. Sci..

[48] Shi C., Wei B., Wei S., Wang W., Liu H., Liu J. (2021). A quantitative discriminant method of elbow point for the optimal number of clusters in clustering algorithm. EURASIP J. Wirel. Commun. Netw..

[49] Goyal M. (2011). Morphological image processing. Int. J. Comput. Sci. Technol..

[50] Webel J., Gola J., Britz D., Mücklich F. (2018). A new analysis approach based on Haralick texture features for the characterization of microstructure on the example of low-alloy steels. Mater. Charact..

[51] Feng X., Gao X., Luo L. (2021). A ResNet50-Based Method for Classifying Surface Defects in Hot-Rolled Strip Steel. Mathematics.

[52] Yang D., Cui Y., Yu Z., Yuan H. (2021). Deep Learning Based Steel Pipe Weld Defect Detection. Appl. Artif. Intell..

[53] Ma B., Wei X., Liu C., Ban X., Huang H., Wang H., Xue W., Wu S., Gao M., Shen Q. (2020). Data augmentation in microscopic images for material data mining. NPJ Comput. Mater..

[54] Ma J., Hu C., Zhou P., Jin F., Wang X., Huang H. (2023). Review of Image Augmentation Used in Deep Learning-Based Material Microscopic Image Segmentation. Appl. Sci..

[55] Li W., Yang L., Peng G., Pang G., Yu Z., Zhu X. (2025). An effective microscopic image augmentation approach. Sci. Rep..

[56] Şengöz N., Yïğ İt T., Özmen Ö., Isik A.H. (2022). Importance of Preprocessing in Histopathology Image Classification Using Deep Convolutional Neural Network. Adv. Artif. Intell. Res..

[57] Zhang J., Li C., Rahaman M., Yao Y., Ma P., Zhang J., Zhao X., Jiang T., Grzegorzek M. (2021). A comprehensive review of image analysis methods for microorganism counting: From classical image processing to deep learning approaches. Artif. Intell. Rev..

[58] Kato S., Hotta K. (2021). Automatic Preprocessing and Ensemble Learning for Low Quality Cell Image Segmentation. arXiv.

